# A retrospective study investigating the anxiety and depression level of novel coronavirus Omicron patients in 2022

**DOI:** 10.1097/MD.0000000000032438

**Published:** 2022-12-23

**Authors:** Yuting Pu, Wen Zhang, Xiangru Xu, Yuting Sun, Caiyu Chen, Shuang Zhou, Bangjiang Fang

**Affiliations:** a LongHua Hospital, Shanghai University of Traditional Chinese Medicine, Shanghai, People’s Republic of China; b Department of Neurology and National Traditional Chinese Medicine Clinical Research Base, the Affiliated Traditional Chinese Medicine Hospital of Southwest Medical University, Luzhou, China; c College of Acupuncture and Massage, Shanghai University of Traditional Chinese Medicine, Shanghai, People’s Republic of China.

**Keywords:** anxiety and depression, protocol, retrospective clinical trial, SARS-CoV-2 Omicron variant

## Abstract

The novel coronavirus disease 2019 continues to spread widely, not only causing physical disorders in patients but also impairing mental health, bringing a heavy burden on global public health. This study aimed to evaluate the anxiety and depression status of patients infected with the severe acute respiratory syndrome coronavirus 2 (SARS-CoV-2) omicron variant in Shanghai.

**Methods/design:**

This study aimed to retrospectively analyze 2000 patients infected with the SARS-CoV-2 Omicron variant. Data from patients assessed with demographic information, anxiety and depressive symptoms were collected using a questionnaire. Clinical and laboratory data were collected using electronic medical system. Anxiety and depression were assessed using the Self-Rating Anxiety Scale, the Generalized Anxiety Disorder Scale, and the Patient Health Questionnaire. Clinical information and laboratory indicators included age, sex, blood pressure, blood glucose, basic disease, time of diagnosis onset, duration of hospitalization, vaccination status of novel coronavirus disease 2019, and virus-negative conversion time.

**Discussion:**

This study will provide evidence-based suggestions for early psychological intervention in patients infected with the SARS-CoV-2 Omicron Variant.

## 1. Introduction

Since the outbreak of the novel coronavirus disease 2019 (COVID-19) in December 2019, virulent strains have been mutating, seriously disrupting people’s daily life.^[1,2]^ The severe acute respiratory syndrome coronavirus 2 (SARS-CoV-2) Omicron variant strain was first reported in south 2021 in November.^[3]^ After 1 year, it has not only become the major variant of COVID-19 worldwide^[4,5]^ but also the dominant strain in the outbreak of COVID-19 in March 2022, Shanghai, China, with a high ability to spread and a faster rate of transmission, causing enormous damage to public health.^[6,7]^ In the face of an ongoing epidemic, patients’ lives may also be adversely affected by quarantine and isolation measures, and community stigmatization, seriously affecting the mental health of infected individuals and the way they interact with others.^[8]^ Individuals infected with the SARS-CoV-2 Omicron Variant may experience varying degrees of psychological crisis, and the persistence of adverse emotions may aggravate patients’ clinical symptoms to some extent and affect their clinical outcomes. This study aimed to retrospectively analyze the relationship between anxiety and depression in patients with the SARS-CoV-2 Omicron Variant in Shanghai and virus-negative conversion time.

## 2. Methods and designs

### 2.1. Study design

This was a retrospective cross-sectional observational study conducted in 5 cabin hospitals, including the Shanghai New International Expo Center, Shanghai City Footprint quarantine venue, Shanghai Songjiang District No. 1 and No. 2 quarantine venue, and the Xinqiao quarantine venue. The study protocol is available at http://www.chictr.org.cn (China Clinical Trials Registry ID: ChiCTR2200063956).

### 2.2. Objectives

This study aimed to retrospectively evaluate the relationship between anxiety and depression in patients with the SARS-CoV-2 Omicron Variant and the time of virus turned negative.

### 2.3. Population

**Figure 1.**
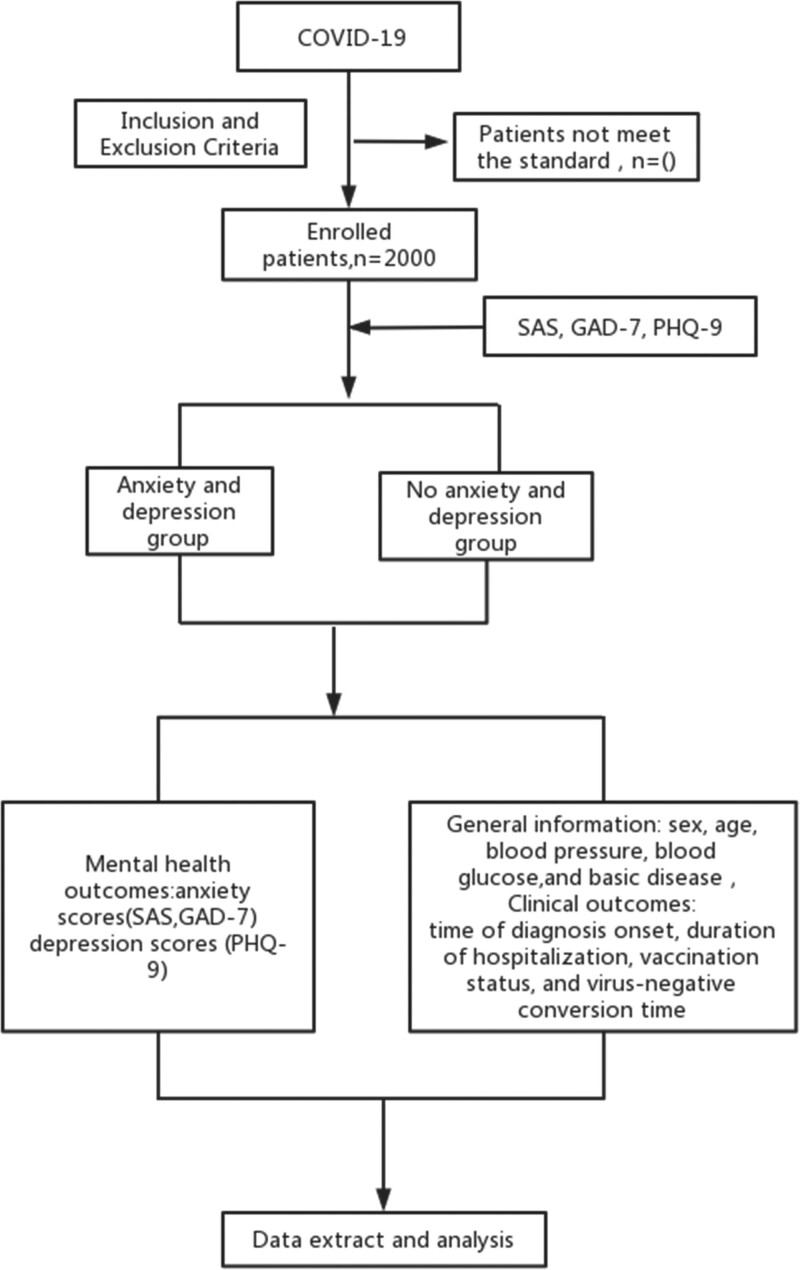
Flowchart of study design.

A total of 2000 patients diagnosed with mild COVID-19 were enrolled from April 1, 2022, to June 15, 2022. The flowchart of the study is shown in Figure 1.

### 2.4. Inclusion criteria

People comply with the diagnostic criteria for mild COVID-19 according to the diagnostic criteria of “ Pneumonia Treatment Protocol for Novel Coronavirus Infection (Trial Version 9)” issued by the National Health Commission of the People’s Republic of China. The patient’s virus was detected as an Omicron variant.

### 2.5. Exclusion criteria

Patients were transferred to another hospital or died between April 1, 2022, and June 15, 2022. Pregnant or breastfeeding women. Patients with other systemic/mental disorders, persistent malignancies, or those undergoing mental health treatment. Patients who did not complete a basic socio-demographic questionnaire.

### 2.6. Outcomes

#### 2.6.1. Anxiety.

Self-rating anxiety scale (SAS): anxiety was assessed by Zung’s Self-Rating Anxiety Scale.^[9]^ The Chinese version of the SAS is widely used in China, and its reliability has been established (Cronbach alpha = .85).^[10]^ The SAS includes 20 items rated on a 4-point Likert scale from 1 (never) to 4 (often), which could be seen in additional file 3, http://links.lww.com/MD/I207. A total score of 1.25 is the final standard score. An SAS score ≥ 50 indicated that the patient had anxiety, with 50 to 59 indicating mild anxiety, 60 to 69 being moderate anxiety, and 70 or more being severe anxiety.

Generalized anxiety disorder scale (GAD-7): the GAD-7 (additional file 4, http://links.lww.com/MD/I208) was also used to assess the severity of anxiety.^[11]^ The tool consists of 7 items rated on a 4-point Likert scale from 0 (not at all) to 3 (nearly every day). Several validation studies have detected cutoff points of ≥6, ≥10, and ≥20, indicating mild, moderate, moderately severe, and severe depression, respectively.^[12]^

#### 2.6.2. Depression.

Patient health questionnaire (PHQ): The PHQ (additional file 5, http://links.lww.com/MD/I210) was used to assess the degree of depression. The questionnaire consisted of 9 items, each with a score ranging from 0 to 3 (0 = not at all, 1 = a few days, 2 = more than half a day, and 3 = almost every day), for a total score of 27. (A score of 6–9 indicates mild depression, 10–14 standing for moderate depression, 15–21 indicates moderately severe depression, and 22–27 indicates severe depression.)^[13,14]^

#### 2.6.3. General information.

Sex, age, blood pressure, blood glucose, and basic disease.

#### 2.6.4. Clinical information and laboratory indicators.

Time of diagnosis onset, duration of hospitalization, vaccination status for COVID-19, and virus-negative conversion time.

### 2.7. Data collection and management

Clinical data were collected using an electronic medical system and questionnaire platform (https://www.wjx.cn/app/survey.aspx). The survey included demographics (participants’ name, gender, age, blood pressure, blood glucose, basic disease) and clinical and laboratory information (time of diagnosis onset, duration of hospitalization, basic disease, vaccination status of COVID-19, virus negative conversion time, SAS, GAD-7, and PHQ-9). The data collected from the questionnaire platform were checked individually, and abnormal data such as confusing answer logic and short answer time were deleted. 10% of the entire sample was randomly selected and checked against in the electronic medical system, with a compliance rate of 85% or more considered acceptable. Excel 2013 was used for data management, and the data were entered by 2 researchers according to the records of the electronic medical system and compared with the data from the questionnaire platform to avoid errors. The aggregated data were then provided to a statistical expert for the statistical analysis. All patients’ personal and medical information will be kept confidential. The results of the statistical analysis will be presented through publications in professional journals or websites.

### 2.8. Statistical analysis

All data were analyzed using the IBM SPSS Statistics software (version 25.0, IBM Corp). Data were expressed as the mean ± standard deviation ($\bar{X}\pm SD$) or median and 95% confidence interval. When the data were normally distributed and the variance was unity, an independent sample *t* test was used to compare the difference between the 2 groups; otherwise, the Mann-Whitney *U* test was used. Multivariate logistic regression was used to analyze the correlation between potential risk factors for anxiety and depression and nucleic acid conversion in COVID-19 patients. The 2-sided test level α = 0.05. *P* value < .05 is considered significant statistically.

### 2.9. Quality control

All the researchers will undergo rigorous training in accordance with the study protocol. The participants will be screened according to the inclusion and exclusion criteria. We will then provide a consent form that includes the participant’s personal information, the purpose of the research, the use and storage of the data, the participant’s responsibilities, and any potential benefits or risks, and they could withdraw from the study at any time before submitting the questionnaire.

### 2.10. Ethics

This trial adhered to the Helsinki Declaration and the Chinese Good Clinical Practice guidelines. The study protocol (version1.0, July 8 2022) was approved by the Medical Ethics Committee of the Jiujiang Hospital of Traditional Chinese Medicine(ethics number: JJSZYY 20220416). If there is any change in the research plan or violation of confidentiality regulations, we will submit it to the Ethics Committee for reexamination. We also registered with the Chinese Clinical Trial Registry (ChiCTR2200063956). Registered on September 21, 2022.

## 3. Discussion

It is well known that altered mental health status is closely related to the body’s immunity and disease recovery.^[15–17]^ However, facing the large-scale outbreak of COVID-19 in Shanghai, most studies have focused on the epidemiological and clinical characteristics of infected patients as well as the genomic signature of the virus and clinical interventions.^[18–21]^ Few studies focused on the mental health status of infected patients. Therefore, this study aimed to analyze the mental health status of patients infected with the SARS-CoV-2 Omicron Variant in Shanghai and its relationship with clinical outcomes.

Multiple studies have revealed that patients infected with the SARS-CoV-2 Omicron Variant experience significant psychological and social stress during isolation treatment and after recovery. On the physical side, they may face changes in their physical functions and illness-related sequelae, such as intermittent fever,^[22]^ pulmonary fibrosis,^[23]^ neurological sequelae,^[24]^ and post-traumatic stress symptoms.^[25]^ On the social side, they may face blame, guilt, and stigma associated with COVID-19.^[26,27]^ These tremendous psychological and social pressures can lead to a bad psychological state, manifesting sleep disorders, anxiety, depression, and even mental illness, seriously affecting the lives of infected individual.^[28]^

Patients infected with the SARS-CoV-2 Omicron Variant are prone to mental health problems. The aggravation of depression and anxiety may lead to the activation of inflammation and a decline in the immune state, even leading to adverse events, which may delay disease recovery. Therefore, we should actively focus on and intervene early in the mental health of patients infected with the SARS-CoV-2 Omicron Variant to improve the psychological and physical status of the diagnosed patients.

## Acknowledgments

We would like to express our appreciation to all medical centers, researchers, and patients, and thank them for their cooperation.

## Author contributions

**Formal analysis:** Yuting Pu, Wen Zhang.

**Investigation:** Shuang Zhou, Bangjiang Fang.

**Methodology:** Xiangru Xu, Caiyu Chen.

**Supervision:** Xiangru Xu, Yuting Sun.

**Writing – original draft:** Yuting Pu, Wen Zhang.

**Writing – review & editing:** Yuting Pu, Bangjiang Fang.

## Supplementary Material
