# Supplementary material for: A retrospective study investigating the anxiety and depression level of novel coronavirus Omicron patients in 2022
Source: Medicine (Baltimore). 2022 Dec 23;101(51):e32438. doi: 10.1097/MD.0000000000032438 (PMC9794253; doi:10.1097/MD.0000000000032438)
Supplement: Supplementary file 1 [file medi-101-e32438-s001.pdf]

Table1. Self-Rating Anxiety Scale score

Self-Rating Anxiety Scale-20 items (SAS)				
**Over the last 2 weeks, how often have you been bothered by any of the following problems?**	**Not at all**	**Several days (1-5 days)**	**More than half the days (6-10 days)**	**Nearly every day (11-14 days)**
1.I feel more nervous and anxious than usual (anxiety)	1	2	3	4
2. I was fear for no reason (fear)	1	2	3	4
3. I get upset easily or feel panicky (panicky)	1	2	3	4
4. I think I might go crazy (insane)	1	2	3	4
5. I think everything is fine and nothing unfortunate will happen (unfortunate premonition)	1	2	3	4
6. My hands and feet are shaking and trembling (trembling hands and feet)	1	2	3	4
7. I struggle with headaches, neck pain and back pain (somatic pain)	1	2	3	4
8. I feel easily debilitated and tired (lethargy)	1	2	3	4
9. I feel calm and easy to sit quietly (meditation is not possible)	1	2	3	4
10. I feel a rapid heartbeat (palpitations)	1	2	3	4
11. I was distressed by a bout of dizziness (dizziness)	1	2	3	4
12. I have fainting episodes or feel like I'm going to faint (fainting sensation)	1	2	3	4
13. I breathe in and out very easily(difficult to breathe)	1	2	3	4
14. My hands and feet are numb and tingling(tingling in my hands and feet)	1	2	3	4
15. I am suffering from stomach pain and indigestion (stomach pain or indigestion)	1	2	3	4
16. I often have to urinate (frequent urination)	1	2	3	4
17. My hands are often dry and warm (sweaty)	1	2	3	4
18. I'm flushed and hot (facial flushing)	1	2	3	4
19. I fall asleep easily and sleep well through the night (sleep disorder)	1	2	3	4
20. I have nightmares (bad dreams)	1	2	3	4
