# Supplementary material for: A retrospective study investigating the anxiety and depression level of novel coronavirus Omicron patients in 2022
Source: Medicine (Baltimore). 2022 Dec 23;101(51):e32438. doi: 10.1097/MD.0000000000032438 (PMC9794253; doi:10.1097/MD.0000000000032438)
Supplement: Supplementary file 2 [file medi-101-e32438-s002.pdf]

Table 2. **Generalized Anxiety Disorder – 7 items (GAD-7)**

**Generalized Anxiety Disorder – 7 items (GAD-7)**				
**Over the last 2 weeks, how often have you been bothered by any of the following problems?**	**Not at all (0 days)**	**Several days (1-5 days)**	**More than half the days (6-10 days)**	**Nearly every day (11-14 days)**
1. Feeling nervous, anxious or on edge?	0	1	2	3
2. Not being able to stop or control worrying?	0	1	2	3
3. Worrying too much about different things?	0	1	2	3
4. Trouble relaxing?	0	1	2	3
5. Being so restless that it is hard to sit still?	0	1	2	3
6. Becoming easily annoyed or irritable?	0	1	2	3
7. Feeling afraid as if something awful might happen?	0	1	2	3
