# Supplementary material for: A retrospective study investigating the anxiety and depression level of novel coronavirus Omicron patients in 2022
Source: Medicine (Baltimore). 2022 Dec 23;101(51):e32438. doi: 10.1097/MD.0000000000032438 (PMC9794253; doi:10.1097/MD.0000000000032438)
Supplement: Supplementary file 3 [file medi-101-e32438-s003.pdf]

Table3: PHQ-9 scores

Patient Health Questionnaire- 9 items (PHQ-9)				
**Over the last 2 weeks, how often have you been bothered by any of the following problems?**	**Not at all (0 days)**	**Several days (1-5 days)**	**More than half the days (6-10 days)**	**Nearly every day (11-14 days)**
1. Little interest or pleasure in doing things?	0	1	2	3
2. Feeling down, depressed, or hopeless?	0	1	2	3
3. Trouble falling or staying asleep, or sleeping too much?	0	1	2	3
4. Feeling tired or having little energy?	0	1	2	3
5. Poor appetite or overeating?	0	1	2	3
6. Feeling bad about yourself - or that you are a failure or have let yourself or your family down?	0	1	2	3
7. Trouble concentrating on things, such as reading the newspaper or watching television?	0	1	2	3
8. Moving or speaking so slowly that other people could have noticed?	0	1	2	3
9. Thoughts that you would be better off dead, or of hurting yourself in some way?	0	1	2	3
